# Monoclinic polymorph of 2-(pyrimidin-2-ylsulfan­yl)acetic acid

**DOI:** 10.1107/S1600536811000651

**Published:** 2011-01-12

**Authors:** Manuela Ramos Silva, Pedro S. Pereira Silva, Consuelo Yuste, José A. Paixão

**Affiliations:** a CEMDRX, Physics Department, University of Coimbra, P-3004-516 Coimbra, Portugal

## Abstract

The title compound, C_6_H_6_N_2_O_2_S, is a new polymorphic form of 2-(pyrimidin-2-ylsulfan­yl)acetic acid. Unlike the previous orthorhombic polymorph [Pan & Chen (2009 ▶) *Acta Cryst.* E**65**, o652], the mol­ecules are not planar: the aromatic ring makes an angle of 80.67 (17)° with the carboxyl plane. In the crystal, mol­ecules are linked by O—H⋯N hydrogen bonds into chains along [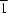
02].

## Related literature

For the previously reported orthorhombic polymorph, see: Pan & Chen (2009 ▶).
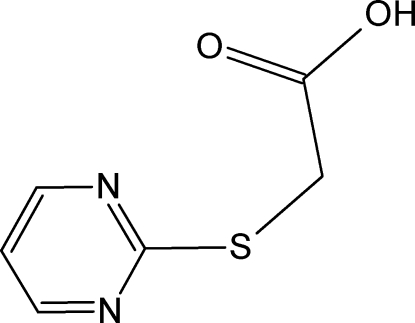


## Experimental

### 

#### Crystal data

C_6_H_6_N_2_O_2_S
*M* *_r_* = 170.19
Monoclinic, 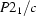

*a* = 8.2617 (3) Å
*b* = 10.3028 (4) Å
*c* = 9.9289 (3) Å
β = 119.845 (2)°
*V* = 733.05 (4) Å^3^
*Z* = 4
Mo *K*α radiation
μ = 0.39 mm^−1^
*T* = 293 K
0.40 × 0.23 × 0.20 mm

#### Data collection

Bruker APEX CCD area-detector diffractometer
Absorption correction: multi-scan (*SADABS*; Sheldrick, 2000 ▶) *T*_min_ = 0.891, *T*_max_ = 0.999
14257 measured reflections
1688 independent reflections
1532 reflections with *I* > 2σ(*I*)
*R*_int_ = 0.027

#### Refinement

*R*[*F* ^2^ > 2σ(*F* ^2^)] = 0.054
*wR*(*F* ^2^) = 0.133
*S* = 1.17
1688 reflections
100 parameters
H atoms treated by a mixture of independent and constrained refinement
Δρ_max_ = 0.31 e Å^−3^
Δρ_min_ = −0.18 e Å^−3^

### 

Data collection: *SMART* (Bruker, 2003 ▶); cell refinement: *SAINT* (Bruker, 2003 ▶); data reduction: *SAINT*; program(s) used to solve structure: *SHELXS97* (Sheldrick, 2008 ▶); program(s) used to refine structure: *SHELXL97* (Sheldrick, 2008 ▶); molecular graphics: *ORTEPII* (Johnson, 1976 ▶); software used to prepare material for publication: *SHELXL97*.

## Supplementary Material

Crystal structure: contains datablocks global, I. DOI: 10.1107/S1600536811000651/bt5454sup1.cif

Structure factors: contains datablocks I. DOI: 10.1107/S1600536811000651/bt5454Isup2.hkl

Additional supplementary materials:  crystallographic information; 3D view; checkCIF report

## Figures and Tables

**Table 1:** Hydrogen-bond geometry (Å, °)

*D*—H⋯*A*	*D*—H	H⋯*A*	*D*⋯*A*	*D*—H⋯*A*
O2—H2⋯N2^i^	0.74	1.97	2.700 (3)	166

Symmetry code: (i) 
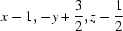
.

**Fig. 1.**
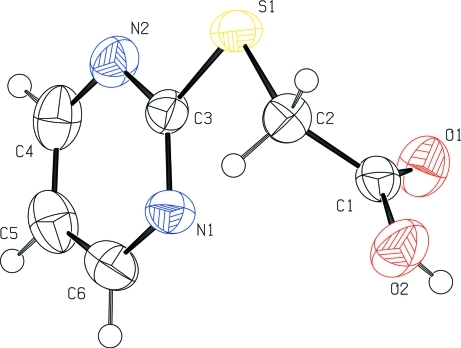
ORTEPII (Johnson, 1976) plot of the title compound. Displacement ellipsoids are drawn at the 50% level.

**Fig. 2.**
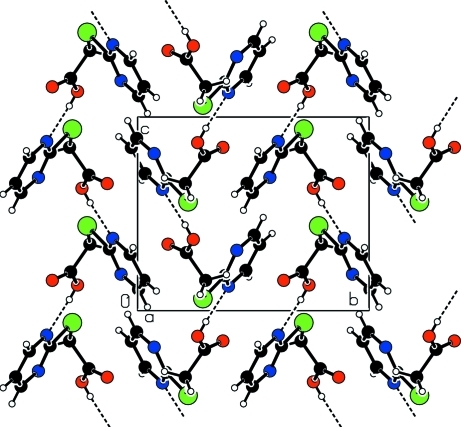
Packing of the molecules in the unit cell showing the H-bonds as dashed lines.

## supplementary crystallographic information

### Comment

In an attempt to synthesize low dimensional compounds with transition metal elements, crystals of a new monoclinic phase of the title compound (Fig. 1) were obtained. In the previously reported orthorhombic phase the molecules are planar and pack in layers, joined by a O—H···N intermolecular bond (Pan & Chen, 2009). In the new monoclinic phase the molecules are not planar, the C3—S1—C2—C1 torsion angle is 77.84 (18)°. There is a similar strong hydrogen bond (Table 1) as in the orthorhombic phase, but in the title compound this intermolecular bond groups the molecules in chains, that run along the [-102] direction.

### Experimental

0.2 mmol of 2-(Pyrimidin-2-ylsulfanyl)acetic acid (98%) and 0.2 mmol of CuCl_2_.2H_2_O (99.0%)were dissolved in 20 ml of water plus 20 ml of ethanol. The solution was slightly warmed and left to evaporate for a few weeks. After that time, small yellowish single crystals were obtained.

### Refinement

Hydrogen atoms bound to C atoms were positioned geometrically with C—H = 0.93–0.97 Å, and constrained to ride on their parent atoms, with *U*_iso_(H) = 1.2Ueq(C). The coordinates of the H atom bound to O were freely refined  with *U*_iso_(H) = 1.5Ueq(O).

### Crystal data

**Table:**

C_6_H_6_N_2_O_2_S	*F*(000) = 352
*M**_r_* = 170.19	*D*_x_ = 1.542 Mg m^−^^3^
Monoclinic, *P*2_1_/*c*	Mo *K*α radiation, λ = 0.71073 Å
*a* = 8.2617 (3) Å	Cell parameters from 6019 reflections
*b* = 10.3028 (4) Å	θ = 2.8–27.2°
*c* = 9.9289 (3) Å	µ = 0.39 mm^−^^1^
β = 119.845 (2)°	*T* = 293 K
*V* = 733.05 (4) Å^3^	Prism, yellow
*Z* = 4	0.40 × 0.23 × 0.20 mm

### Data collection

**Table:**

Bruker APEX CCD area-detector diffractometer	1688 independent reflections
Radiation source: fine-focus sealed tube	1532 reflections with *I* > 2σ(*I*)
graphite	*R*_int_ = 0.027
φ and ω scans	θ_max_ = 27.6°, θ_min_ = 2.8°
Absorption correction: multi-scan (*SADABS*; Sheldrick, 2000)	*h* = −10→10
*T*_min_ = 0.891, *T*_max_ = 0.999	*k* = −13→13
14257 measured reflections	*l* = −12→12

### Refinement

**Table:**

Refinement on *F*^2^	Primary atom site location: structure-invariant direct methods
Least-squares matrix: full	Secondary atom site location: difference Fourier map
*R*[*F*^2^ > 2σ(*F*^2^)] = 0.054	Hydrogen site location: inferred from neighbouring sites
*wR*(*F*^2^) = 0.133	H atoms treated by a mixture of independent and constrained refinement
*S* = 1.17	*w* = 1/[σ^2^(*F*_o_^2^) + (0.0619*P*)^2^ + 0.3572*P*] where *P* = (*F*_o_^2^ + 2*F*_c_^2^)/3
1688 reflections	(Δ/σ)_max_ < 0.001
100 parameters	Δρ_max_ = 0.31 e Å^−^^3^
0 restraints	Δρ_min_ = −0.18 e Å^−^^3^

### Special details

**Table:**

Geometry. All e.s.d.'s (except the e.s.d. in the dihedral angle between two l.s. planes) are estimated using the full covariance matrix. The cell e.s.d.'s are taken into account individually in the estimation of e.s.d.'s in distances, angles and torsion angles; correlations between e.s.d.'s in cell parameters are only used when they are defined by crystal symmetry. An approximate (isotropic) treatment of cell e.s.d.'s is used for estimating e.s.d.'s involving l.s. planes.
Refinement. Refinement of *F*^2^ against ALL reflections. The weighted *R*-factor *wR* and goodness of fit *S* are based on *F*^2^, conventional *R*-factors *R* are based on *F*, with *F* set to zero for negative *F*^2^. The threshold expression of *F*^2^ > σ(*F*^2^) is used only for calculating *R*-factors(gt) *etc*. and is not relevant to the choice of reflections for refinement. *R*-factors based on *F*^2^ are statistically about twice as large as those based on *F*, and *R*- factors based on ALL data will be even larger.

### Fractional atomic coordinates and isotropic or equivalent isotropic displacement parameters (Å^2^)

**Table:**

	*x*	*y*	*z*	*U*_iso_*/*U*_eq_	
S1	0.79236 (8)	0.78200 (6)	0.43768 (7)	0.0451 (2)	
O2	0.2618 (3)	0.7405 (2)	0.1323 (3)	0.0642 (6)	
H2	0.2106	0.7051	0.0572	0.096*	
O1	0.5054 (3)	0.63141 (18)	0.1564 (2)	0.0544 (5)	
C1	0.4398 (3)	0.7107 (2)	0.2002 (3)	0.0383 (5)	
C2	0.5444 (3)	0.7939 (2)	0.3434 (3)	0.0411 (5)	
H2A	0.5048	0.7701	0.4171	0.049*	
H2B	0.5094	0.8838	0.3146	0.049*	
C3	0.8442 (3)	0.8782 (2)	0.3184 (2)	0.0358 (5)	
N1	0.7074 (2)	0.92846 (19)	0.1883 (2)	0.0407 (4)	
C6	0.7635 (4)	1.0039 (2)	0.1100 (3)	0.0495 (6)	
H6	0.6732	1.0425	0.0185	0.059*	
C5	0.9468 (4)	1.0271 (3)	0.1575 (3)	0.0544 (7)	
H5	0.9820	1.0810	0.1013	0.065*	
C4	1.0766 (4)	0.9677 (3)	0.2916 (3)	0.0536 (7)	
H4	1.2028	0.9803	0.3261	0.064*	
N2	1.0268 (3)	0.8926 (2)	0.3736 (2)	0.0459 (5)	

### Atomic displacement parameters (Å^2^)

**Table:**

	*U*^11^	*U*^22^	*U*^33^	*U*^12^	*U*^13^	*U*^23^
S1	0.0382 (3)	0.0520 (4)	0.0360 (3)	−0.0017 (2)	0.0116 (2)	0.0089 (2)
O2	0.0394 (10)	0.0662 (13)	0.0664 (13)	0.0044 (8)	0.0108 (9)	−0.0106 (10)
O1	0.0514 (10)	0.0554 (11)	0.0595 (11)	−0.0035 (8)	0.0299 (9)	−0.0133 (9)
C1	0.0362 (11)	0.0383 (12)	0.0411 (12)	−0.0037 (8)	0.0197 (9)	0.0045 (9)
C2	0.0398 (12)	0.0454 (13)	0.0418 (12)	−0.0021 (9)	0.0233 (10)	−0.0007 (10)
C3	0.0341 (10)	0.0336 (11)	0.0360 (11)	0.0000 (8)	0.0147 (9)	−0.0030 (8)
N1	0.0371 (9)	0.0426 (11)	0.0396 (10)	0.0039 (8)	0.0169 (8)	0.0058 (8)
C6	0.0604 (15)	0.0433 (14)	0.0478 (13)	0.0062 (11)	0.0291 (12)	0.0072 (11)
C5	0.0705 (17)	0.0440 (14)	0.0667 (17)	−0.0068 (12)	0.0477 (15)	−0.0022 (12)
C4	0.0437 (13)	0.0522 (15)	0.0729 (18)	−0.0101 (11)	0.0350 (13)	−0.0138 (13)
N2	0.0325 (9)	0.0479 (12)	0.0511 (12)	−0.0016 (8)	0.0161 (9)	−0.0032 (9)

### Geometric parameters (Å, °)

**Table:**

S1—C3	1.753 (2)	C3—N2	1.333 (3)
S1—C2	1.783 (2)	N1—C6	1.335 (3)
O2—C1	1.313 (3)	C6—C5	1.365 (4)
O2—H2	0.7449	C6—H6	0.9300
O1—C1	1.177 (3)	C5—C4	1.370 (4)
C1—C2	1.510 (3)	C5—H5	0.9300
C2—H2A	0.9700	C4—N2	1.328 (3)
C2—H2B	0.9700	C4—H4	0.9300
C3—N1	1.327 (3)		
			
C3—S1—C2	102.08 (10)	N2—C3—S1	113.08 (16)
C1—O2—H2	109.4	C3—N1—C6	114.8 (2)
O1—C1—O2	125.2 (2)	N1—C6—C5	123.2 (2)
O1—C1—C2	126.1 (2)	N1—C6—H6	118.4
O2—C1—C2	108.7 (2)	C5—C6—H6	118.4
C1—C2—S1	115.15 (16)	C6—C5—C4	117.1 (2)
C1—C2—H2A	108.5	C6—C5—H5	121.5
S1—C2—H2A	108.5	C4—C5—H5	121.5
C1—C2—H2B	108.5	N2—C4—C5	121.6 (2)
S1—C2—H2B	108.5	N2—C4—H4	119.2
H2A—C2—H2B	107.5	C5—C4—H4	119.2
N1—C3—N2	126.8 (2)	C4—N2—C3	116.5 (2)
N1—C3—S1	120.15 (16)		
			
O1—C1—C2—S1	8.2 (3)	C3—N1—C6—C5	0.8 (4)
O2—C1—C2—S1	−172.38 (16)	N1—C6—C5—C4	0.8 (4)
C3—S1—C2—C1	77.84 (18)	C6—C5—C4—N2	−1.2 (4)
C2—S1—C3—N1	−4.9 (2)	C5—C4—N2—C3	0.0 (4)
C2—S1—C3—N2	174.94 (16)	N1—C3—N2—C4	1.8 (4)
N2—C3—N1—C6	−2.2 (3)	S1—C3—N2—C4	−177.99 (18)
S1—C3—N1—C6	177.60 (17)		

### Hydrogen-bond geometry (Å, °)

**Table:**

*D*—H···*A*	*D*—H	H···*A*	*D*···*A*	*D*—H···*A*
O2—H2···N2^i^	0.74	1.97	2.700 (3)	166

Symmetry codes: (i) *x*−1, −*y*+3/2, *z*−1/2.
